# Influenza antivirals and animal models

**DOI:** 10.1002/2211-5463.13416

**Published:** 2022-04-27

**Authors:** C. Joaquin Caceres, Brittany Seibert, Flavio Cargnin Faccin, Stivalis Cardenas‐Garcia, Daniela S. Rajao, Daniel R. Perez

**Affiliations:** ^1^ 1355 Department of Population Health College of Veterinary Medicine University of Georgia Athens GA USA

**Keywords:** animal models, antiviral‐host factors, antivirals, influenza, mice, neuraminidase inhibitors

## Abstract

Influenza A and B viruses are among the most prominent human respiratory pathogens. About 3–5 million severe cases of influenza are associated with 300 000–650 000 deaths per year globally. Antivirals effective at reducing morbidity and mortality are part of the first line of defense against influenza. FDA‐approved antiviral drugs currently include adamantanes (rimantadine and amantadine), neuraminidase inhibitors (NAI; peramivir, zanamivir, and oseltamivir), and the PA endonuclease inhibitor (baloxavir). Mutations associated with antiviral resistance are common and highlight the need for further improvement and development of novel anti‐influenza drugs. A summary is provided for the current knowledge of the approved influenza antivirals and antivirals strategies under evaluation in clinical trials. Preclinical evaluations of novel compounds effective against influenza in different animal models are also discussed.

## Graphical abstract

Influenza A and B viruses are among the most prominent human respiratory pathogens, with 3–5 million severe cases annually. Antivirals are part of the first line of defense against influenza. This review summarizes the current understanding of antivirals that have been approved and those in clinical trials, and preclinical antivirals that are being studied in the context of different animal models.

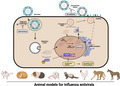

**Abbreviations**
BXM: baloxavir marboxil
CEN: cap‐dependent endonuclease
DANA: 2,3‐dehydro‐2‐deoxy‐N‐acetylneuraminic acid
EIV: equine IAV
GSH: golden Syrian hamsters
HA: hemagglutinin
HPAI: highly pathogenic avian influenza
IAV: Influenza A
IBV: influenza B
LPAI: low pathogenicity avian influenza viruses
NA: neuraminidase
NAC: N‐acetyl cysteine
NAI: neuraminidase inhibitors
NHC: N4‐hydroxycytidine
NHP: non‐human primates
NP: nucleoprotein
PA: polymerase acidic protein
PB1: polymerase basic protein 1
PB2: polymerase basic protein 2
pdmH1N1: pandemic H1N1 IAV
PR8: A/Puerto Rico/8/34
RDN: Reduning
RdRp: RNA‐dependent RNA polymerase
ROS: reactive oxygen species
vRNPs: viral ribonucleoprotein particles
x31: A/HKx31

## Introduction

Influenza A virus (IAV) and influenza B virus (IBV) infections cause between 3 and 5 million severe cases with 300 000–650 000 deaths every year. IAV and IBV are enveloped viruses containing 8 segments of single‐strand negative‐sense genomic RNA in the form of viral ribonucleoprotein particles (vRNPs) associated with the polymerase complex (PB1, PB2, PA) and the nucleoprotein (NP) [1]. On the virus’ surface, IAV and IBV display two major glycoproteins called hemagglutinin (HA) and neuraminidase (NA). Antigenic differences in the HA and NA of IAVs allow for further classification of these viruses into subtypes H1‐H18 and N1‐N11. In humans, H1N1 and H3N2 subtype IAVs and two antigenically distinct IBV lineages (Victoria and Yamagata) are responsible for seasonal influenza epidemics. HA and NA are under constant immune pressure leading to antigenic drift that enables evasion of the host antibody responses [2]. Antigenic drift in the HA is one of the main reasons for the need to update vaccines and re‐vaccination. In addition to HA and NA, IAV and IBV contain the integral membrane M2 protein. IBV also contains a fourth surface protein called NB. Both M2 and NB contain ion channel activity [1].

Along with vaccination, the use of complementary antiviral strategies can reduce the overall disease burden caused by influenza infections. Antivirals help with viral clearance and reduce transmission and deaths associated with influenza infections. Currently, FDA‐approved antiviral treatments designed specifically against influenza include M2 inhibitors such as adamantanes (rimantadine and amantadine), neuraminidase inhibitors (NAI; peramivir, zanamivir, and oseltamivir), and more recently the cap‐dependent endonuclease inhibitor targeting the PA polymerase subunit (baloxavir) [3, 4]. In this review, we summarize information regarding the use of the currently approved antivirals and those under clinical trials. We summarize also the preclinical evaluation of alternative antivirals and the variety of animal models used for such purposes. Finally, we discuss host determinants and host responses that have been evaluated under antiviral treatment.

## FDA approved antivirals

### Adamantanes

**Fig. 1:**
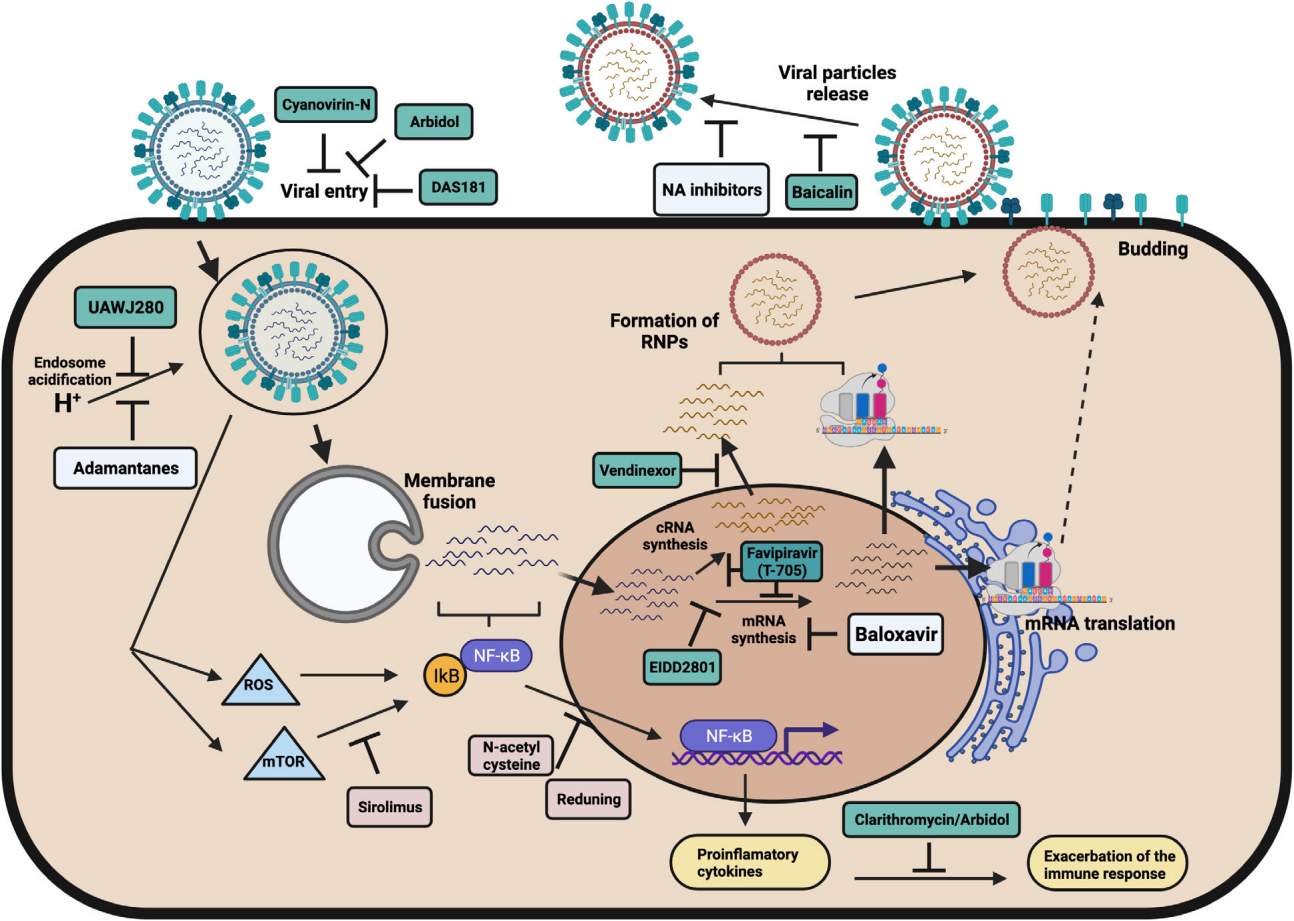
Influenza replication steps inhibited by influenza antivirals. Schematic representation of the influenza replicative cycle showing the steps inhibited by FDA‐approved influenza antivirals (light blue), antiviral under evaluation in clinical trials (light red), and antivirals under preclinical evaluation (cyan). Figure created with BioRender.com.

The influenza M2 ion channel is essential for virus replication by acting as a proton channel that acidifies the virus’ interior during the transition in the endosome leading to the release of the vRNPs into the cytoplasm (Fig. 1). The vRNPs subsequently migrate to the nucleus where viral transcription and replication take place. The high degree of amino acid conservation of the M2 channel made it an attractive target for antiviral development [5]. Two antiviral drugs: amantadine and rimantadine demonstrated that blocking the M2 channel, resulted in inhibition of viral uncoating and viral replication (Table 1). These drugs were the first approved antivirals against influenza for clinical use in 1966 and 1993, respectively [6, 7]. M2 inhibitors showed antiviral activity when human subjects naturally infected with H1N1 were treated therapeutically. Subjects under treatment with either one of these drugs showed reduced fever and shed less virus than the placebo‐treated group [8]. Several studies were performed to assess the safety and efficacy among different doses of amantadine (50, 100, and 200 mg·day^−1^). The results showed that 100 and 200 mg·day^−1^ prevented illness, decreased virus replication, and decreased the infection rate in individuals challenged intranasally with a prototypic H3N2 IAV strain. Side effects associated with neurological signs were observed in individuals treated with 200 mg·day^−1^, but not 100 mg·day^−1^ [9]. Further reports of clinical trials worldwide demonstrated the efficacy of amantadine upon infection with different H1N1 and H3N2 IAV strains. Adamantanes at daily prophylactic doses of 100 mg were the most effective at preventing influenza infections in human subjects [6, 10].

**Table 1:** Currently FDA‐approved antivirals against influenza.

Class of antiviral	Name	Commercial name	Year of approval	Administration route	Treatment regime	Mutations associated with resistance	% Of resistant strains circulating	References
M2 inhibitors	Amantadine	Symmetrel^a^	1966	Oral	200 mg·day^−1^ in 1 or 2 divided doses	M2 (L26F, V27A, A30V, S31N, G34E, and L38F)	99.9% (H1N1 and H3N2 strains) 45.2% (H1‐H17) 13% H5N1	[6, 7, 8, 14, 16, 17]
	Rimantadine	Flumadine	1993	Oral	100 mg twice a day for 7 days			
NA inhibitors	Oseltamivir	Tamiflu	2016	Oral	75 mg twice a day for 5 days	NA (H274Y, I222R/K/V, G146R, E119A/G/D, Q136K, R292K, N295S, V116A, and H117V)	1.3 ‐ 3.2% (H1N1) and < 1% (H3N2 and IBV)	[21, 25, 26, 27, 38, 39]
	Peramivir	Rapivab	2014	Intravenous	600mg, single dose within 2 days after onset of symptoms			
	Zanamivir	Relenza	1999	Inhalation	10 mg twice a day for 5 days			
PA inhibitor	Baloxavir	Xofluza	2018	Oral	40mg (weight < 80 kg) or 80 mg (weight > 80 kg) one dose	PA (I38T, I38F, and I38M, E23G/K, A37T, and E199G)	9.7% after baloxavir treatment	[4, 48, 49, 50, 52, 53, 58]

^a^ Discontinued.

M2 inhibitors have become increasingly obsolete. IBVs are naturally resistant to M2 inhibitors and in IAVs M2 inhibitor resistance increased from 0.4% in the 1994–1995 season to 12.3% in the 2003‐2004 season worldwide [11, 12, 13]. The widespread circulation of resistant strains associated with M2 mutations such as L26F, V27A, A30V, S31N, G34E, and L38F have made M2 inhibitors ineffective [14]. Among H1N1 IAVs, almost 9000 out of 12 000 strains identified from human subjects around the world were found to contain amantadine‐resistant mutations, most notably after the emergence of the 2009 pandemic H1N1 IAV (pdmH1N1). In the early 2000s, samples from IAV positive amantadine‐treated children showed a high frequency of the M2 amino acid mutations S31N and A30V in H3N2 IAVs, and V27A in H1N1 IAVs. At least 80% of the children shed virus with adamantane‐resistant mutations [14, 15]. In the United States, > 96% of H3N2 IAVs circulating in the 2005–2006 season were adamantane resistant. Currently, > 99% of circulating human IAVs carry adamantane‐resistant mutations prompting the CDC to not recommend the use of M2 inhibitors for the treatment of influenza [16, 17].

Analyses of 31 251 M2 protein sequences selected from IAVs of human, avian, swine, equine, and canine origin spanning the period from 1902 to 2013, showed that 45.8% of these sequences contained amino acid signatures predictive of adamantane resistance (Table 1) [14]. Eurasian origin highly pathogenic avian influenza (HPAI) H5N1 IAVs isolated between 2002 and 2012 showed an increased frequency of resistance to adamantanes. H5N1 IAVs carrying double mutations in M2 such as the L26I and S31N mutations or mutations in positions V27A or S31N has been linked to amantadine resistance [16, 17]. In the H7 subtype IAVs, resistance to amantadine have been detected in strains from China, United States, Pakistan, and Italy. In the United States and Italy, H7N1 and H7N2 HPAI strains, respectively, were associated with the M2 S31N amantadine‐resistant marker [14]. Amantadine was not effective in inhibiting virus replication in MDCKs of an H7N7 HPAI strain, nor protecting mice or reducing viral titers postchallenge [18]. Interestingly, the H7N7 HPAI virus did not harbor any of the known M2 mutations associated with amantadine resistance. It was argued that the H7 HA protein overcomes the effects of amantadine on M2 in the H7N7 strains [18]. Increased amantadine resistance has also been observed in poultry‐adapted Eurasian origin low pathogenic avian influenza viruses of the H9 subtype, linked to mutations such as V27A, A30T, S31N [14, 16, 17, 19]. It is tempting to speculate that increased amantadine resistance in poultry origin endemic IAV strains is the direct result of the unapproved use of amantadine.

### NA inhibitors

It is commonly accepted that NA sialidase activity is relevant early in influenza infection acting on mucopolysaccharides to allow the virus to attach to target cells and late in the infection during virus budding on the viral HA allowing for the release of discrete virus particles. Zanamivir (Relenza), oseltamivir (Tamiflu), and peramivir (Rapivab) block the NA catalytic site affecting virus entry and release (Fig. 1). Zanamivir was developed based on knowledge of the analog 2,3‐dehydro‐2‐deoxy‐N‐acetylneuraminic acid (DANA) as a weak NA inhibitor. Further substitution of the C4‐OH with a 4‐gianidino group generated zanamivir with a 10 000‐fold binding enhancement to NA. Oseltamivir and peramivir are structurally like zanamivir with side‐chain modifications introducing a pentyl ether side chain instead of the glycerol side chain and a C4 amino group in the case of oseltamivir and a hydrophobic side chain instead of the glycerol side chain and a C4‐guanidino substitution for peramivir [20]. Zanamivir is administered through an inhaler 10 mg twice daily for 5 days as the standard treatment. Oseltamivir is administered orally 75 mg twice daily for 5 days. And for peramivir, a single dose of 600 mg intravenously is recommended within 2 days after onset of symptoms for treatment of uncomplicated influenza (Table 1). Due to ease of administration, oseltamivir is currently the standard of care for the treatment of influenza. Oseltamivir is recommended for patients with severe influenza illness and/or patients at risk for influenza complications such as asthma, immunosuppression, obesity, and children younger than two years or elderly among others [21]. Several studies have shown that NAIs are effective at decreasing influenza‐related complications and mortality when administered within 2 days after the onset of clinical signs. However, in most of the cases, NAIs are administered after 48 h decreasing their efficacy [22, 23, 24]. The effect of NAIs has been demonstrated in H1N1 and H3N2 IAV subtypes [25, 26]. NAIs are also effective against IBV infections but with lower efficacy in comparison to IAV. Zanamivir is more effective against IBV than oseltamivir [27]. Factors such as gender, antibiotic treatment, and age have been associated with the effectiveness of NAI treatment [28].

Neuraminidase inhibitors have been evaluated to treat zoonotic infections with the Guangdong‐lineage HPAI H5N1 virus. Improvement in survival was observed among H5N1 infected patients treated with oseltamivir compared to those without treatment [29, 30]. More recent zoonotic HPAIV H5N6 strains have been shown susceptible to NAIs [31, 32]. In contrast, more recent HPAIV H5N8 has shown reduced susceptibility to NAIs [33, 34, 35]. In the case of zoonotic infections with H7N9 viruses from China, cases of reduced susceptibility have been observed, which correlate with poor clinical outcomes [36, 37].

Mutations in NA conferring NAI resistance are particularly problematic in immunocompromised patients. NAI‐resistant strains readily emerge during NAI treatment of severe or fatal cases of influenza [38]. Different mutations have been characterized to confer NAI resistance in which H274Y (N2‐numbering throughout the text) is one of the most prevalent. The H274Y mutation plus changes in position 222 (I222R/K/V) or G146R further reduce NAI susceptibility [38, 39]. Additionally, NAI‐resistant mutations including E119G/V (H1N1), E119A/V (H7N9), R292K (H7N9), N294S (H1N1), V116A (H1N1), H117V (H1N1), and I117V (H5N1) have been reported in different subtypes [40, 41, 42, 43, 44]. A study with 67 H5N1 isolates found ~ 13% of NAI‐resistant variants (Table 1). Mutations in the NA enhancing the resistance to NAI in IBV are also present such as D197Y or V430I [45, 46].

## Baloxavir and favipiravir to inhibit viral polymerase activity

The RNA‐dependent RNA polymerase (RdRp) complex in influenza is comprised of three subunits, the polymerase basic protein 2 (PB2), the polymerase basic protein 1 (PB1), and the polymerase acidic protein (PA) that form a heterotrimer essential for virus transcription and replication. Transcription of viral RNAs depends on a ‘cap snatching’ mechanism that utilizes the cap‐binding activity of PB2 and the cap‐dependent endonuclease (CEN) in the PA subunit [1]. Structural studies revealed that specific residues in the N‐terminus of PA were highly conserved and mutations in these residues resulted in the loss of endonuclease activity [4, 47]. Through computer‐aid design, baloxavir marboxil (BXM, XofluzaTM, formerly S‐033188) was developed to specifically block PA’s CEN activity (Fig. 1). Initially, in vitro studies demonstrated that BXM blocked CEN activity without affecting PB2 or PB1 activities with 2‐3 log10 lower EC_90_ values than approved NAIs [4, 48]. BXM was shown to have a broad antiviral effect against an array of IAV of various subtypes and IBV, including those with NAI‐resistant mutations [4, 48, 49, 50]. BXM is administered orally as a single dose of the prodrug of baloxavir acid (S‐033447; BXA) with a half‐life of ~ 79 h (Table 1) [51]. Phase three clinical trials showed that a single dose of BXA was safe, superior to placebo in relieving influenza symptoms, and more efficient at reducing viral titers 1 day after treatment in uncomplicated and high‐risk influenza patients [52, 53]. In 2018, BXA/BXM was approved for human use in Japan and the United States for the treatment of uncomplicated influenza in adults and adolescents (> 12 years), who have been symptomatic for < 48 h. In 2019, FDA approval for BXA/BXM was expanded to include patients at high risk of developing influenza‐related complications [51, 54]. Previous studies also showed that combinations of BXA/BXM and either NAIs or favipiravir had increased antiviral effects against pdmH1N1 and H3N2 IAVs compared with single‐drug treatment [55].

BXM‐resistant variants emerge readily during treatment with up to 120‐fold reduced drug susceptibility (Table 1) [56, 57]. The PA I38T mutation has been shown to increase resistance to BXM *in vitro* and *in vivo* [58]. *In vitro* studies showed impaired replication fitness of variants carrying either the PA I38T, I38F, or I38M in H1N1 and H3N2 viruses while only the PA I38F was impaired in IBV. Additional PA mutations (E23G/K, A37T, and E199G) have been identified *in vitro* to have a significant but lesser impact on BXM susceptibility [59, 60]. Variants harboring the PA I38T, I38F, or I38M mutations were prevalent in 2.2–9.7% of BXM recipients during phase 2 and phase 3 trials resulting in the initial delay of influenza symptoms but accompanied by prolonged virus shedding and rise in viral titers [52, 53]. In a different trial, variants with PA I38X substitutions emerged after 3‐day post‐treatment and were detected until 9 days after treatment in 9.7% of patients, in which 85.3% of patients had increased virus titers [58]. Like previous reports associated with oseltamivir resistance, the rate of PA I38X variants was higher in children < 5 years of age treated with BXM [54, 61]. Like adults, pediatric patients demonstrated increased symptom duration time, higher frequency of fever recurrence, and prolonged virus detection [54, 62, 63]. A more recent study showed that H1N1 and H3N2 IAV variants with reduced susceptibility to BXM have similar replication and transmissibility to wild‐type viruses in ferrets yet the substitution compromises the replication fitness of H1N1 viruses in vitro [64].

An alternative antiviral is favipiravir, a nucleoside analog that targets the RdRp and inhibits viral RNA synthesis by terminating elongation and thus impeding the synthesis of complementary viral RNAs [65]. *In vitro* studies showed reduced plaque formation after favipiravir treatment against laboratory‐adapted and clinical isolates of influenza A, B, and C viruses [66]. Favipiravir has been tested in clinical trials for seasonal influenza in Japan and the United States [65], and it was approved for treatment as a countermeasure for novel and re‐emerging influenza viruses in Japan in 2014 [65]. A recent study assessed the efficacy of favipiravir as a combination therapy with oseltamivir in patients in China with severe influenza resulting in accelerated clinical recovery [67]. While not isolated in a clinical setting, favipiravir‐resistant viruses produced by reverse genetics carry the K229R mutation in the PB1 gene; however, the PB1 K229R variant virus was not dominant in cell culture [65]. Nevertheless, the PB1 K229R variant virus was shown successfully infect ferrets and transmit via direct contact and respiratory droplets, yet the frequency of the K229R mutation decreased over time *in vivo* [68].

## Antivirals under clinical trials

As of October 2021, there are 15 clinical trials underway that involve the use of antiviral compounds for the treatment of influenza‐associated illnesses (*ClinicalTrials.gov*, Table 2). These trials include treatments with Baloxavir, N‐acetyl cysteine, Sirolimus or Rapamycin, Zanamivir, ZSP1273, Reduning, HEC116094, GP681, TG‐1000, and Oseltamivir. Oseltamivir is included in 10 of these studies either as a drug under investigation (NCT04515446), as a combination therapy (NCT03900988, NCT03901001, NCT04327791, NCT04712539, and NCT04982913), and/or as an active comparator (NCT03754686, NCT03900988, NCT03901001, NCT04327791, NCT05012189, NCT04712539, NCT04683406, NCT04183725, and NCT04982913) (Table 2). Novel antiviral compounds/interventions for which public information is available are described below.

**Table 2:** Antivirals currently under clinical trials. IAV, influenza A virus; IBV, influenza B virus; ICU, intensive care unit; LRTI, lower respiratory tract infection; SARI, severe acute respiratory infection; URTI, upper respiratory tract infection. Source: ClinicalTirals.gov.

Antiviral	Title	Trial #	Target population	Outcome measurements	Phase	Research group/institution	Locations
Oseltamivir	Quantification of Viral Load in the Upper Respiratory Tract in Patients Treated with Oseltamivir for Influenza	NCT04515446	In‐patient adults (≥ 18 years) admitted for influenza and treated with oseltamivir	*Primary*: Duration of virus shedding by type IAV or IBV *Secondary*: Duration of virus shedding by vaccination status and comorbidities. Difference in number of days in isolation between hospitalize patients treated with oseltamivir	N/A	Centre Hospitalier Princesse Grace	Monaco
Oseltamivir vs. Paracetamol	Oseltamivir Versus Paracetamol for Influenza‐like Illness During the Influenza Season	NCT03754686	Adults (≥ 18 years) hospitalized during high season with SARI	*Primary*: Failure to reach clinical stability, transfer to ICU, readmission within 30 days or death within 30 days *Secondary*: Time to clinical stability and duration of hospitalization	IV	Rambam Health Care Campus	N/A
N‐acetyl cysteine + Oseltamivir or 5% Dextrose + Oseltamivir	Intravenous N‐acetylcysteine and Oseltamivir Versus Oseltamivir in Adults Hospitalized with Influenza and Pneumonia	NCT03900988	Adults hospitalized with influenza complicated by LRTI	*Primary*: Normalization of respiratory status in days (oxygen saturation and respiratory rate) *Secondary*: Copies of viral RNA. Levels of interleukins 6, 8, 17, and 18, chemokine ligand 9, sTNFR‐1, CRP, phospho‐p38 and phospho‐ERK, phospho‐inhibitor kB/IkB. Resolution of symptoms in days. ICU admission. Mortality in days. Incidence of treatment‐emergent adverse events	IV	Chinese University of Hong Kong	N/A
Sirolimus + Oseltamivir vs. Oseltamivir	Adjunctive Sirolimus and Oseltamivir Versus Oseltamivir Alone for Treatment of Influenza	NCT03901001	Adults (≥ 18 years) positive for IAV or IBV and hospitalized with complicated LRTI	*Primary*: Normalization of respiratory status in days (oxygen saturation and respiratory rate) *Secondary*: Copies of viral RNA. Levels of interleukins 6, 8, 17, and 18, chemokine ligand 9, sTNFR‐1, CRP, phospho‐p38 and phospho‐ERK, phospho‐inhibitor kB/IkB. Resolution of symptoms in days. ICU admission. Mortality in days. Incidence of treatment‐emergent adverse events	IV	Chinese University of Hong Kong	N/A
Baloxavir	Study to Assess the Safety, Pharmacokinetics, and Efficacy of Baloxavir Marboxil in Healthy Pediatric Participants from Birth to < 1 Year with Influenza‐Like Symptoms	NCT03653364	Infants (≤ 1 year) positive and symptomatic for influenza infection	*Primary*: % of participants with side effects and severe side effects *Secondary*: Drug pharmacokinetics. Time to alleviation of influenza signs and symptoms. Duration of fever and symptoms. Time to return to normal health and activity. Frequency of influenza‐related complications. % Requiring antibiotics. Duration of virus shedding. Virus titers	III	Hoffman‐La Roche	USA, Costa Rica, Mexico, Finland, Israel, Panama, Poland, Russia South Africa, Spain, Thailand
Baloxavir vs. Placebo	Study to Assess the Efficacy of Baloxavir Marboxil Versus Placebo to Reduce Onward Transmission of Influenza A or B in Households	NCT03969212	Individuals (5–64 years) positive for influenza virus and negative for SARS‐CoV‐2. Leaving in a household with people negative for influenza or SARS‐CoV‐2	*Primary*: Virus transmission by day 5 postrandomization *Secondary*: Virus and symptomatic transmission by day 5 and 9 postrandomization. % of index patients with adverse effects. Change in productivity and activity impairment in index patients only	III	Hoffman‐La Roche	USA, Argentina, Brazil, Bulgaria, Chile, China, Costa Rica, France, Greece, Hong Kong, Hungary, India, Israel, Japan, Mexico, New Zealand, Poland, Singapore, South Africa, Spain, Turkey, UK
Oseltamivir + Baloxavir vs. Oseltamivir + Placebo	Combination Therapy with Baloxavir and Oseltamivir 1 for Hospitalized Patients with Influenza (The COMBO Trial 1)	NCT04327791	Adults (≥ 18 years) with laboratory confirmed IAV or IBV infection	*Primary*: Time to clearance of viral shedding	II, III	Bassett Healthcare; Genentech, Inc.; Viroclinics Biosciences B.V.	USA
Baloxavir vs. Oseltamivir	Baloxavir Versus Oseltamivir for Nursing Home Influenza Outbreaks	NCT05012189	Adult (≥ 18 years) nursing home residents and staff	*Primary*: Number of ILI cases after randomization *Secondary*: Outbreak duration. Number of antiviral course treatments needed to control the outbreak per facility. Respiratory‐related hospitalizations in residents ≥ 65 years old during influenza season 2021‐2021 after randomization. Mortality rate after randomization	IV	Insight Therapeutics, LLC; Brown University; Case Western Reserve University; Genentech, Inc.	USA
Baloxavir + Oseltamivir vs. Oseltamivir	Baloxavir and Oseltamivir for the Treatment of Severe Influenza Infection in Immunocompromised Patients	NCT04712539	Influenza‐positive patients, recipient of hematopoietic cell transplants or have a hematological malignancy. Evidence of LRTI or high risk of URTI	*Primary*: Changes in viral loads. Incidence of complicated hospital stay *Secondary*: Antiviral resistance rate. Progression to LRTI. Length of hospital stay. Oxygen requirement. Rate of respiratory failure. Morality rate. Changes in microbiome diversity	II	M.D. Anderson Cancer Center	USA
ZSP1273 + Oseltamivir placebo vs. Oseltamivir + ZSP1273 placebo	A Study of ZSP1273 Tablets in Patients with Acute Uncomplicated Influenza A	NCT04683406	Adults (≥ 18 to ≤ 64 years) positive for influenza infection	*Primary*: Time to alleviation of symptoms *Secondary*: Antiviral resistance rate. % of participants with virus titers. % of participants with detectable viral RNA. Duration of virus shedding. % of participants with alleviated symptoms. Time to alleviation of symptoms. % of participants with influenza‐related complications	III	Guangdong Raynovent Biotech Co., Ltd	China, at 73 locations
Reduning + Oseltamivir Phosphate granules simulants vs. Oseltamivir Phosphate granules + Reduning simulants	Clinical Study of Reduning Injection for the Treatment of Influenza in Children	NCT04183725	Children (2–14 years) positive for influenza	*Primary*: Time to temperature recovery *Secondary*: Time to alleviation of symptoms. Degree of disease remission. Rate of negative for influenza testing. Number and frequency of antipyretic/analgesic drugs used. Incidence of complications	IV	China Academy of Chinese Medical Sciences; Children's Hospital of Soochow University; Anhui Provincial Children's Hospital; Qilu Children's Hospital of Shandong University; Tianjin 4th Centre Hospital; Renmin Hospital of Wuhan University; Hebei Maternity&Child Healthcare Hospital; The Second Affiliated Hospital of Jiaxing University; The First Hospital of Hunan University of Chinese Medicine; Affiliated Hospital of Shanxi University of Traditional Chinese Medicine	N/A
HEC116094 vs. Oseltamivir vs. HEC11609 + Oseltamivir	Single Dose and Multiple Dose Safety, Tolerability, PK and Food Effect Study and Interaction with Oseltamivir Study of HEC116094 in Healthy Adult Subjects	NCT04982913	Healthy adults (18–45 years)	*Primary*: Safety and tolerability. *Secondary*: Drug pharmacokinetics	I	Sunshine Lake Pharma Co., Ltd.	China
Zanamivir	An Intravenous (IV) Zanamivir Pharmacokinetics (PK) Study in Hospitalized Neonates and Infants with Influenza Infection	NCT04494412	Neonates and infants (≤ 6 months) with influenza infection	*Primary*: Drug pharmacokinetics. *Secondary*: Adverse effects. Number of participants with abnormal findings in temperature, respiratory rate, oxygen saturation, heart rate. Number of participants with phenotypic and genotypic resistance. Number of patients with resistance emergence. Virus load over time	II	Glaxo Smith Kline	Poland, Spain
GP681 tables vs. GP681 simulants	A Clinical Study on the Safety and Efficacy of GP681 Tablets in the Treatment of Uncomplicated Acute Influenza	NCT04736758	Adults (18–65 years) with symptomatic influenza infection	*Primary*: Time to alleviation of influenza symptoms	II	Jiangxi Qingfeng Pharmaceutical Co. Ltd.	China
TG‐1000 vs. Placebo	To Evaluate the Efficacy and Safety of TG‐1000 Compared with Placebo in Adult Patients with Acute Uncomplicated Influenza Virus Infection	NCT04706468	Adults (18–65 years) with uncomplicated influenza infection	*Primary*: Drug antiviral activity vs the placebo *Secondary*: Time to alleviation of symptoms. % Of patients positive for viral RNA. Time to resolution of fever. Time to return to preinfluenza status. Body temperature. Incidence of influenza‐related complications	II	TaiGen Biotechnology Co.; Ltd. R&G Pharma Studies Co., Ltd.	China

*N‐acetyl cysteine* (NAC) is a mucolytic agent that is used to treat mucus secretions. NAC is also a potent antioxidant that stimulates glutathione biosynthesis and enhances glutathione S‐transferase activity. NAC is approved for the treatment of acetaminophen toxicity. NAC has been shown to reduce mucus secretory cell hyperplasia in a rat model for pulmonary fibrosis [69], likely by reducing oxidative stress. Influenza virus infection induces the production of reactive oxygen species (ROS), which in turn activates NF‐κB thereby upregulating proinflammatory cytokines and chemokines [70]. ROS, in particular hydroxyl radical (OH^‐^), is associated with pulmonary edema during acute lung injury [71]. A549 lung carcinoma cells infected with a prototypic highly pathogenic H5N1 IAV and treated with NAC resulted in reduced virus replication, reduced apoptosis, and reduced production of proinflammatory responses associated with inhibition of NF‐κB and protein kinase p38 [72]. NAC has a very short plasma half‐life, ~ 2.5 to ~ 5.7 h after oral or intravenous administration, respectively [73, 74]. Administration of high doses (1000 mg·kg^−1^) of NAC in combination with oseltamivir (1 mg·kg^−1^) has been shown to protect mice from lethal influenza challenge [75]. A phase IV clinical trial is currently evaluating the use of N‐acetyl cysteine (NAC) in combination with Oseltamivir, compared to treatment with Oseltamivir alone (NCT03900988, Table 2) to determine whether NAC provides additional benefits in the clinical management of influenza patients with lower respiratory tract involvement and abnormal respiratory status.

*Sirolimus (rapamycin)* is a macrocyclic antibiotic originally isolated from *Streptomyces hygroscopicus,* with potent activity against *Candida* sp and antitumor and immunosuppressive properties [76, 77, 78, 79]. Rapamycin immunosuppressive activity depends on the binding to immunophilins (cytosolic peptidyl isomerases), specifically FKBP12 in the family of FK506 binding proteins (FKBPs) [80]. The rapamycin/FKBP12 complex binds the kinase known as the target of rapamycin (TOR, mTOR in mammals), a member of the phosphatidylinositol 3‐kinase‐related kinase family of protein kinases. Binding of the rapamycin/FKBP12 complex to TOR/mTOR inhibits cell proliferation by interfering with cell cycle progression from G1 to S phase in T cells, carcinogenic cells, and other cell types [81]. Rapamycin was found to have potent activity against lymphocytic leukemia, B16 melanocarcinoma, brain ependymoblastoma, mammary, and colon tumors (reviewed in [82]). The use of rapamycin as a treatment against influenza clinical illness has been explored in animal models and in clinical trials. Results from mouse studies have shown contrasting results. In one study rapamycin in combination with oseltamivir treatment of H1N1 infected mice led to reduced virus replication, reduced lung pathology [83], and linked to reduced activation of mTOR and suppression of inflammasome‐mediated cytokine production [83]. A different study, however, showed no antiviral activity and even exacerbated clinical outcomes in mice infected with either H1N1 or H3N2 IAVs [84, 85]. In humans, rapamycin treatment in combination with corticosteroids in patients with severe H1N1‐associated pneumonia and on mechanical ventilator support, led to improved arterial oxygen partial pressure to fractional inspired oxygen (PaO_2_/FiO_2_) ratios, reduced organ failure assessment scores, shorter duration of ventilator use and faster virus clearance, compared to treatment with corticosteroids alone [86]. Rapamycin in combination with oseltamivir is set for evaluation in a randomized clinical trial in hospitalized patients with influenza‐related illness (NCT03901001, Table 2).

### Reduning injection

Reduning (RDN) is a traditional Chinese medicine preparation, composed of *Lonicera japonica* Thunb., *Gardenia jasminoides* Ellis, and *Artemisia annua* L. RDN has anti‐inflammatory properties associated with inhibition of the AMPK/MAPK/NF‐κB signaling pathway [87]. Studies in mice and rats with LPS‐induced lung injury suggested that RDN can downregulate the expression of proinflammatory cytokines IL‐1β, IL‐6 and TNF‐α; decreased the expression of LPS‐inducible nitric oxide synthase [88, 89]. A multiscale modeling analysis, integrating molecular docking, network pharmacology, and the clinical symptoms information, was performed to explore the interaction mechanism of RDN on human diseases such as influenza infections [90]. Multiple potential protein targets for the RDN components were identified associated with proinflammatory responses [90]. Studies in mice found that RDN treatment provided protection against influenza mortality ranging between 10% and 37% depending on the strain. The combination of RDN with ribavirin increased the survival of infected mice, associated with decreased production of reactive oxygen species (ROS) and lung pathology, and decreased proinflammatory cytokines compared with monotherapy or untreated controls [67, 91]. Clinical trials have shown that RDN treatment alleviates symptoms and febrile responses in influenza‐infected individuals. RDN is under a phase IV clinical trial to assess its efficacy for the treatment of influenza in children (NCT04183725, Table 2).

## Antiviral evaluation in animal models of influenza

### Mice

**Fig. 2:**
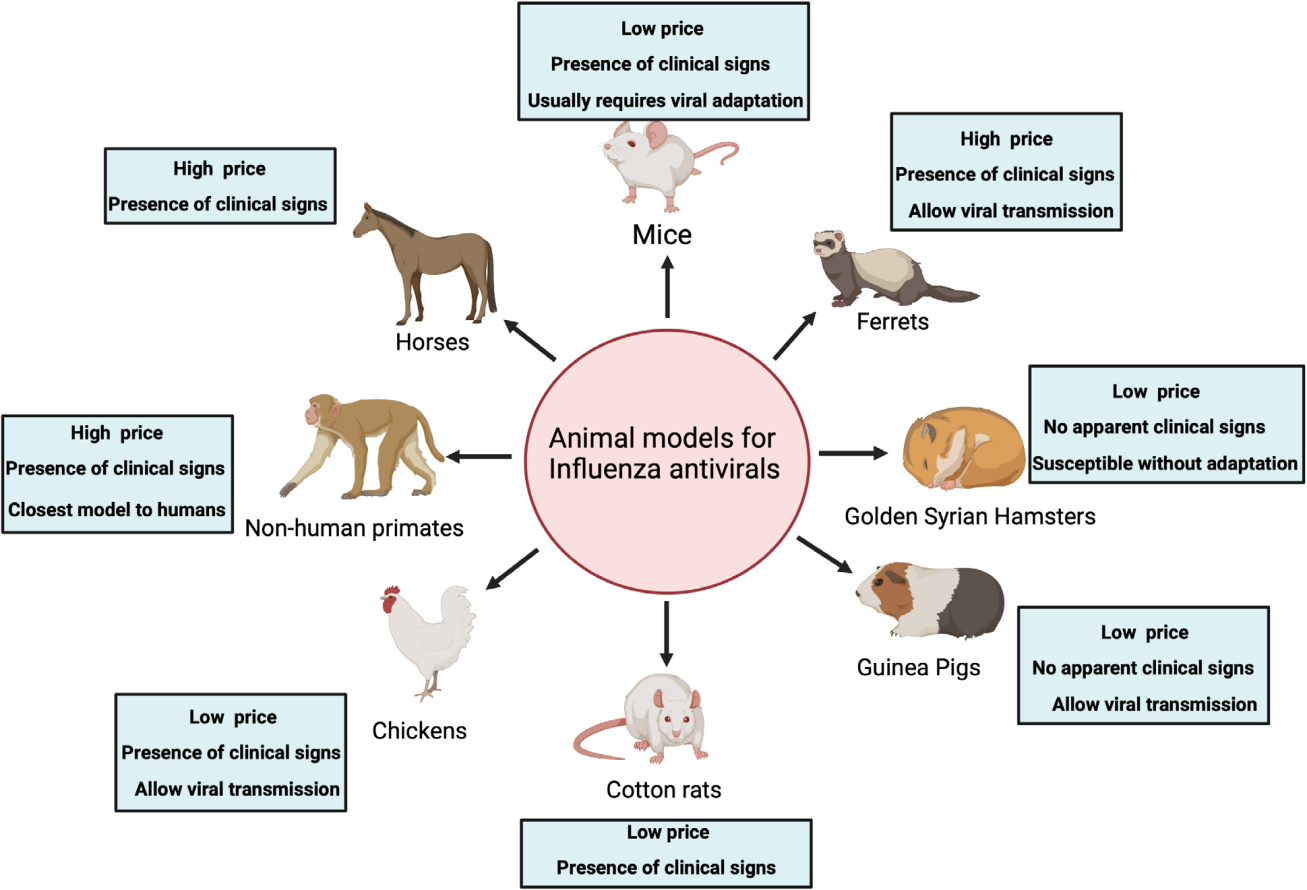
Animal models used for the evaluation of influenza antivirals. Summary of the different animal models used for influenza antivirals. Figure created with BioRender.com.

Mice (*Mus musculus*) are extensively used for influenza research (Fig. 2). A major drawback of this model is that mice are not naturally susceptible to influenza (Table 3). Many influenza strains require adaptation to become infectious to mice. However, for influenza strains that replicate well, the clinical signs that mice experience upon influenza infection (body weight loss, activity, appearance, survival, and occasionally neurological symptoms) and the availability of numerous reagents to study such phenomenon make them excellent models for the evaluation of countermeasures against influenza [92]. Intranasal inoculation of influenza viruses is the most frequent infection route in mice. Meanwhile, oral administration of candidate compounds is the most common route of administration, but other routes such as intraperitoneal, intramuscular, intranasal, and intravenous routes have been used [93].

**Table 3:** Animal models used for antiviral evaluation against influenza. N/R, not reported.

Animal model	Presence of clinical signs	Transmission	References
Mice (*Mus musculus*)	Yes	No	[94, 95, 96, 97, 98, 99, 100, 101, 102, 109, 110, 111]
Golden Syrian Hamster (*Mesocricetus auratus*)	No	Yes	[92, 122]
Cotton rats (*Sigmodon hispidus*)	Yes	No	[123, 124]
Guinea pigs (*Cavia porcellus*)	No	Yes	[117, 126, 142]
Ferret (*Mustela furo*)	Yes	Yes	[144, 145, 146, 147, 148, 149, 150, 151, 152, 154, 155, 156]
Chickens (*Gallus gallus*)	Yes	Yes	[161, 162, 163]
Non‐human primates	Yes	N/R	[31, 146, 165, 166, 167, 168, 169]
Horses (*Equus caballus*)	Yes	N/R	[170, 171, 172]

Amantadine and rimantadine were tested via different routes in Swiss‐Webster and BALB/c mice, respectively, showing protection against influenza. Later, it became well known the mechanism of action, blocking the M2 channel and inhibiting viral replication. Amantadine treatment also protected C57BL/6 mice after infection with a neurovirulent recombinant IAV (R404BP). Oral administration of oseltamivir decreased lung viral titers and increased survival in BALB/c mice infected with H1N1, H3N2, and IBV viruses [94, 95]. In BALB/c mice, zanamivir therapeutic monotherapy showed a significant reduction of mean weight loss against H3N2 virus challenge and decreased mortality, weight loss, and reduced viral titers against H1N1 virus challenge [96]. Peramivir as single or multiple dose intramuscular administration at 24‐ or 48‐h postinfection in BALB/c mice prevented mortality, reduced weight loss, and lung virus titers in mice infected with oseltamivir‐resistant H1N1 virus [97, 98].

Oral administration of the polymerase inhibitor T‐705 (favipiravir) prevented mortality and reduced lung virus titers in BALB/c mice challenged with lethal doses of the laboratory‐adapted A/Puerto Rico/08/1934 (H1N1) virus [65, 66] or oseltamivir‐resistant or sensitive HPAI H5N1 strains [99, 100, 101, 102]. Likewise, oral administration of T‐705 protected C57BL/6 mice in a dose‐dependent manner against oseltamivir‐resistant IBV. When administered orally, BXM showed significant reductions of IAV (H1N1, H5N1, and H7N9) and IBV titers including oseltamivir‐resistant strains within 24 h after treatment [52, 103, 104, 105, 106].

Among preclinical experimental approaches, Baicalin produced from extracts of the traditional Chinese medicinal plant *Scutellaria baicalensis Georgi* demonstrated antiviral activity by inhibiting NA activity. Baicalin administered intravenously partially protected ICR mice challenged with an H1N1 strain [107]. Likewise, berberine, a natural isoquinoline from the *berberis* species, showed antiviral activity in C57BL/6 mice infected with H1N1 reducing viral replication, inflammation, and pulmonary edema. Berberine suppressed the expression of immune‐related molecules such as TLR7, MyD88, and NF‐kb, and inhibited the MAPK/ERK pathway, a signaling process that is essential for the nuclear export of the viral ribonucleoprotein [108]. Therapeutic treatment with a combination of baicalein and ribavirin improved survival rates, body weight loss, and lung inflammatory response of H1N1 challenged‐ICR mice [109]. Influenza‐infected (H1N1 or H3N2) BALB/c mice treated with Verdinexor, a nuclear export inhibitor drug, displayed limited virus shedding, reduced proinflammatory cytokines, and moderated leukocyte lung infiltration. Influenza replication inhibition in the presence of Verdinexor seems to be related to blocking XPO1, a mediator of nuclear export [110]. UAWJ280, a novel deuterium‐containing M2 inhibitor developed against amantadine‐resistant (S31N) strains, was effective in BALB/c mice improving clinical signs and survival, alone or in combination with oseltamivir when administered prophylactically, against lethal challenge with either oseltamivir‐sensitive or ‐resistant H1N1 strains [111]. Prophylactic administration of DAS181, a sialidase fusion protein that removes sialic acid from the epithelium protected BALB/c mice against lethal HPAI H5N1 virus challenge. DAS181 treatment 72‐h postinfection also protected animals challenged with H5N1, H1N1, and H7N9 strains [112, 113, 114, 115]. UV‐4, a novel iminosugar improved survival and clinical signs in Balb/c mice challenged with IAV and IBV strains [116]. Ribonucleoside analogs that get incorporated into viral RNAs, leading to increase viral mutagenesis have also been evaluated. BALB/c mice infected with H1N1 showed a reduction in lung viral loads when animals were prophylactically treated at either 100 or 400 mg·kg^−1^ with *N^4^*‐hydroxycytidine (NHC), which is the active compound of the prodrug EIDD2801. Similar results were observed when mice were infected with HPAI H5N1, proving that the drug may be a promising candidate to treat influenza infections [117].

Influenza antiviral drugs licensed in other countries have also been evaluated in mice. Arbidol hydrochloride, which is licensed in Russia and China for influenza treatment, inhibits HA‐mediated membrane fusion, and therefore, influenza replication showed antiviral activity against H1N1, H3N2, and H9N2 in BALB/c mice following infection. The compound exhibited increased survival rates, inhibited weight loss, and increased the lung index compared with the control group, demonstrating effectiveness in the treatment of influenza infections in the mouse model [118, 119].

### Golden Syrian hamster

Golden Syrian hamsters (GSH; *Mesocricetus auratus*) are naturally susceptible to a wide range of IAV subtypes including H1N1, H2N2, H3N2, H5N1, and H9N2 and IBV (Fig. 2) [92]. Intranasal inoculation is the most common infection route, but intragastric inoculation of H5N1 and aerosol transmission of pandemic H1N1 and seasonal H3N2 IAV have also been reported in GSH [120, 121]. The GSH model has been used to understand influenza virus pathogenesis, transmission, and influenza countermeasures because of its susceptibility to influenza viruses without prior adaptation, ease of handling, and relatively inexpensive maintenance costs [92, 122]. While H3N2 virus replication has been detected in multiple respiratory organs, weight loss and clinical signs are generally not observed during infection; however, various reports suggest weight loss during pandemic H1N1 and BXM‐resistant IAV infections [122]. Major drawbacks of this model are the absence of visible clinical signs during infection despite high viral titers in the respiratory tract and scarce availability of reagents, limiting its use [92, 93, 121].

### Cotton rats

Cotton rats (*Sigmodon hispidus*) are susceptible to IAV and IBV such as seasonal human H3N2 and H1N1 strains and mouse‐adapted strains such as A/Puerto Rico/8/34 (PR8) and A/HKx31 (x31) without prior adaptation. Intranasal inoculation is the most frequent infection route and common clinical signs are an increase in respiratory rate, fever, and weight loss (Fig. 2) [93]. Cotton rats treated with zanamivir did not fare better than untreated controls with respect to distress and lung lesions associated with H3N2 virus infection [123]. Cotton rats challenged with the H3N2 virus and treated with the corticosteroid triamcinolone acetonide, alone or in combination with zanamivir or oseltamivir showed reduced histopathological lesions with suppression of IFN‐ γ response [124].

### Guinea pigs

Guinea pigs (*Cavia porcellus*) are susceptible to numerous IAV subtypes such as H1N1, H3N2, H5Nx, H7N9, and IBV [125, 126, 127, 128, 129, 130, 131, 132, 133, 134, 135, 136, 137, 138, 139, 140] without prior adaptation. Guinea pigs, most commonly the Hartley strain, are utilized as an animal model for influenza research (Fig. 2) [92]. Comparable to golden Syrian hamsters and cotton rats, guinea pigs are susceptible to influenza viruses without prior adaptation, are easier to handle, and possess relatively inexpensive maintenance costs. The guinea pig anatomy and physiology of the lungs resemble that of humans; however, limited reagents specific to guinea pigs and lack of clinical disease signs postinfluenza virus infection, notably in H5N1 infection limit its use [92, 122, 141]. Like other animal models, intranasal inoculation is the most frequent infection route. IAV‐infected guinea pigs treated with N^4^‐hydroxycytidine (NHC) or interferon showed a reduction in viral titers and reduced virus transmission [117, 126, 142]. The guinea pig animal model is most used to examine the potential pathogenicity and transmission of antiviral‐resistant influenza strains, which can have extensive implications for pandemic preparedness [92, 142].

### Ferrets

Ferrets (*Mustela furo*) are naturally susceptible to influenza A and B viruses and present similar characteristics for influenza infection as humans in terms of clinical signs, respiratory tract pathology, virus replication and transmission, and immunity making them an excellent model to study countermeasures against IAV and IBV (Fig. 2). Clinical signs of infected ferrets are like those in humans, including sneezing, nasal secretions, fever, gastrointestinal symptoms such as diarrhea, neurological complications, weight loss, and lethargy. Commonly, ferrets are experimentally infected via intranasal routes leading to viral replication within 8‐day postinfection, but intratracheal inoculation has also been employed [93, 143]. Strains with adamantane resistance readily emerge in influenza‐infected ferrets treated with amantadine. NAI treatment in ferrets provides similar levels of alleviation of clinical signs and lung pathology as seen in humans although such intervention in ferrets is not necessarily correlated with reduction of virus shedding. In general, prophylactic NAI treatment was shown to reduce transmission of pdmH1N1 and HPAI H5N1 in ferrets [92, 93].

More recently, it was shown that combinations studies with zanamivir plus poly‐L‐glutamine have improved antiviral activity as a combination therapy in comparison with zanamivir itself [144]. In addition, peramivir ameliorated disease caused by HPAI H5N1 infection in ferrets by reducing viral titers in the lungs and brain and improving the survival of infected ferrets [145]. Similar results were observed upon infection with IBV with reduced viral titers and clinical signs after a single treatment with peramivir 1‐day postinfection [146]. The efficacy of BXM has also been demonstrated in ferrets as decreased viral shedding in the upper respiratory tract and reduced transmission between ferrets in comparison with the placebo and oseltamivir‐treated group for IAV and IBV [147, 148, 149].

Additionally, multiple drugs under preclinical evaluations have been assessed in ferrets. One of the drugs that has been evaluated is EIDD2801; EIDD2801 demonstrated antiviral activity against pdmH1N1, H3N2 seasonal IAV, and IBV virus when ferrets were treated therapeutically exhibiting decreased viral shedding, airway epithelium histopathology, inflammation, and ameliorated clinical signs [150, 151]. Arbidol hydrochloride showed antiviral activity against different strains of IAV by reducing clinical signs, histopathological lesions, and the activation of proinflammatory cytokines induced by influenza infection [119]. Another compound that showed antiviral activity against pdmH1N1 is nitazoxanide when it is administered in combination with oseltamivir prophylactically decreasing the days of viral shedding [152]. Cyanovirin‐N, which is capable of binding to glycosylation sites in the HA, inhibits influenza virus replication in vitro and showed antiviral activity in ferrets at early timepoints after challenge with a 2‐fold reduction in viral titers when it was administered in a prophylactic regime [153]. Verdinexor, which inhibits the nuclear export of viral ribonucleoprotein and thus inhibits the replication of IAV and IBV, is another effective antiviral against pdmH1N1 in ferrets decreasing viral titers and histopathological lesions in lungs and decreasing the activation of proinflammatory cytokines such as IFN‐ γ and TNF‐ α [110]. Fluororibosides, ribavirin, and cyclooctylamine hydrochloride have been evaluated in ferrets as well [154, 155, 156]. In other cases, no antiviral activity has been observed in ferrets such as in the case of Chloroquine despite the antiviral activity observed in vitro [157].

### Chickens

Chickens (*Gallus gallus*) together with other poultry species such as turkeys or quails are susceptible to different subtypes of IAV. IAVs of avian origin are classified as low pathogenicity avian influenza viruses (LPAI) or HPAI based on the pathotype in chickens and/or the presence of a polybasic amino acid cleavage site composed of arginine (R) or lysine (K). Outbreaks of HPAIs H5Nx or H7Nx or LPAIVs H9N2 are associated with high mortality, delayed growth, and lower eggs production generating a detrimental effect and economical losses in the poultry industry. Additionally, poultry species play an important role in the generation of novel IAV strains with zoonotic potential [158]. Chickens infected with LPAI typically present mild‐to‐severe respiratory diseases such as coughing, sneezing, rales, rattles, ocular discharge, decreased activity, mild weight loss, and occasionally diarrhea. Meanwhile, clinical signs associated with HPAI vary but can include lethargy, swelling of the comb and wattles, cyanosis, neurological signs such as tremors of the head and neck, paralysis, convulsions, loss of balance, or death without prior clinical signs [159]. Infection routes include intranasal, intratracheal, intravenous, and ocular‐nasal routes [160]. Due to the relevance of chickens for IAV, few antivirals have been evaluated in this model against HPAI. BXM and peramivir showed to be effective against H5N6 reducing viral titers and mortality when chickens are immediately treated after the challenge [161]. In the case of H9N2, positive results have been observed with oseltamivir upon challenge with IAV H9N2 or Taishan Pinus massoniana pollen polysaccharides reducing viral loads and histopathological lesions [162, 163].

### Non‐human primates

Non‐human primates (NHP) are another model used to evaluate antiviral compounds against influenza (Fig. 2). NHP resembles humans in terms of genetic, anatomical structures, and immune response. Ethical concerns and husbandry requirements reduce the use of this animal model for the study of influenza (Table 3) [92]. Regarding infection route, intratracheal, intranasal, oral, or intraocular routes have been used individually or simultaneously. Like humans, clinical signs in NHP range from asymptomatic to mild infections, including conjunctivitis, anorexia, and nasal discharge during influenza infection [92, 164]. Zanamivir and peramivir were evaluated in cynomolgus macaques infected with HPAI H5N1 virus resulting in reduced viral loads, reduced body weight loss, reduced proinflammatory cytokine production, and pneumonia in a dose‐dependent manner [165, 166]. Peramivir and oseltamivir have also been evaluated against IBV in cynomolgus macaques leading to reduced viral shedding in the presence of peramivir but not in the case of oseltamivir. Nevertheless, a reduction in the production of proinflammatory cytokines was observed in both groups [146]. Prophylactic treatment with oseltamivir was administered against pdmH1N1 with reduced susceptibility to oseltamivir (NA H274Y) resulting in decreased virus shedding. However, the best outcome was seen in the group challenge with the oseltamivir‐sensitive pdmH1N1 strain [167]. Oseltamivir and peramivir were also effective in decreasing the burden associated with the HPAI H5N6 virus, whereas amantadine did not exhibit antiviral activity [31]. Comparisons of treatments with either BXM, oseltamivir, or zanamivir were also carried out in cynomolgus macaques challenged with the HPAI H7N9 virus; results showed the best outcome in the BXM‐treated animals [168]. Prophylactic clarithromycin, which possesses anti‐inflammatory properties, has also been evaluated against HPAI H5N1 and LPAI H7N9 strains resulting in improvement in clinical signs and decreased viral titers [169].

### Horses

Horses (*Equus caballus*) have been used for the assessment of antivirals against equine IAV (EIV) of the H3N8 and H7N7 subtypes. Clinical manifestation of EIV is associated with fever, cough, and nasal discharge (Table 3). EIV is usually not deadly in horses but may predispose them to secondary infections [170]. Regarding infection route, inoculation using an ultrasonic nebulizer has been used [171]. The effect of peramivir was demonstrated against a panel of 7 different EIVs where milder clinical signs and a decrease in viral shedding were observed. In addition, therapeutic treatment with BXM demonstrated efficacy against EIV; however, viruses carrying mutations in the PA that confers resistance to BXM were detected in nasal washes, like what has been observed in humans [171, 172].

## Modulation of antiviral activity by host factors

Multiple host factors including sex, pregnancy, obesity, age, and underlying respiratory conditions have been shown to affect disease severity during influenza virus infection and thus potentially affect the safety and efficacy of antiviral treatments [173]. Previous reports using murine models suggest that sex hormones may contribute to the disease severity of IAV in female mice compared with male mice. In most mouse models, young adult female mice demonstrate more severe outcomes and increased pulmonary inflammatory response compared with male mice [174]. Further, sex differences were observed when analyzing adverse effects from oseltamivir in rats showing that male rats were more vulnerable to developing adverse effects compared with female rats [174]. When analyzing oseltamivir treatment in humans, alleviation of symptoms and reduction of nasal viral titers were greater in males than females; however, data also suggested that females clear oseltamivir faster than males when analyzed in newborns [28]. It has been suggested that the absorption or metabolism of the antiviral compound contributes to the observed differences in sex as females have greater carboxylesterase 1‐mediated oseltamivir hydrolysis than males [175, 176]. However, sex differences were not observed in reduced symptoms or virus load among females and males treated with zanamivir [28]. In addition to sex, the safety and efficacy evaluation of oseltamivir administration in pregnant women showed that there was no evidence for association between oseltamivir and poor fetal growth and adverse fetal outcomes such as miscarriage and preterm delivery [177]. Further, there was a significantly lower risk of small‐for‐gestational age in the group that used oseltamivir by reducing symptoms of influenza, thus supporting the use of antiviral oseltamivir during pregnancy [177]. Obesity has been associated with a higher risk for severe influenza‐like illness along with decreased immune response during influenza virus infection [173]. Utilizing a genetically obese and diet‐induced mouse model (﻿B6.V‐Lepob/J and ﻿C57BL/6J), it was shown that mortality and lung pathology increased in the obese model; however, oseltamivir treatment enhanced survival [178]. Meanwhile, surveillance studies showed that antiviral therapy significantly decreased hospitalization stays in obese individuals and timely antiviral treatment appears to be an important cofounder among influenza severity and obesity [179]. Lastly, zanamivir had comparable efficacy and reduced pulmonary complications in patients with asthma or chronic pulmonary disease without producing adverse side effects [180]. Taken together, antiviral use, particularly oseltamivir and zanamivir, has been demonstrated to be safe and improve disease severity in populations that are at a higher risk of developing the severe disease during influenza infection.

## Conclusions

Influenza antivirals together with vaccines are the first line of defense against IAV and IBV infections. Current FDA‐approved antivirals have been demonstrated to be effective against different subtypes of IAV and IBV. However, concerns have been raised about the acquisition of resistant mutation against the different antivirals, which is more evident in the adamantanes where 99% of the influenza strains circulating are resistant to this class kind of antivirals. Due to the need for the development of novel influenza antivirals, several studies under the clinical or preclinical phase have been reported. Multiple animal models have been used for preclinical evaluation but predominantly mice and ferrets. Additionally, host factors such as sex, pregnancy, and obesity possess an effect on the antiviral efficacy.

## Author contributions

CJC, BS, FCF, SCG, DSR, and DRP compiled the information relevant to this review, generated tables, and wrote the first version of the manuscript. CJC and DRP edited and wrote the final version of the manuscript. CJC generated figures.

## Conflict of interest

The authors declare no conflict of interest.
